# Sleep patterns among middle school students: a three-year longitudinal study in the context of China’s “Double Reduction” policy

**DOI:** 10.3389/fpubh.2025.1594904

**Published:** 2025-10-29

**Authors:** Jiansong Dai, Yan Huang, Yifan Zhao, Kai Xu, Zhongke Gu, Gangrui Chen

**Affiliations:** ^1^Department of Sports and Health Sciences, Nanjing Sport Institute, Nanjing, China; ^2^Department of Graduate, Nanjing Sport Institute, Nanjing, China; ^3^Sports Science Research Institute, Nanjing Sport Institute, Nanjing, Jiangsu, China

**Keywords:** adolescent sleep, sleep pattern, “Double Reduction” policy, wearable device, sleep deprivation

## Abstract

**Objective:**

This longitudinal study aims to describe the sleep pattern trajectories among Chinese middle school students over a three-year period.

**Methods:**

From December 2021 to December 2023, 82 middle school students were monitored for 2 weeks per academic year for a period of 3 years using the Huawei Band 6 wearable devices, and sleep parameters were collected at each stage and statistically analyzed.

**Results:**

For 82 middle school students, their sleep onset time gradually delayed over the years, leading to a yearly decrease in night sleep duration (Grade 7: 452.3 ± 32.4 min; Grade 8: 432.9 ± 38.6 min; Grade 9: 420.4 ± 37.9 min, *F* = 50.30, *p*<0.001). The incidence of sleep deprivation gradually increased (*x*^2^ = 17.09, *p* = 0.0002). Over the 3 years, there were no statistically significant differences in wake-up time, sleep onset time, or night sleep duration between genders, and there was no interaction with time and gender. Sleep duration on rest days was significantly longer than on school days (*p* < 0.001). There was a significant interaction between time and time type (rest day/school day) on daytime sleep duration, wake-up time, and sleep onset time (*p* < 0.05).

**Conclusion:**

This study indicates that middle school students experience a gradual decrease in sleep duration over 3 years. Schools are advised to delay morning start times and enhance sleep hygiene education for students and parents as part of measures to address insufficient sleep among students.

## 1 Introduction

During adolescence, significant physiological changes, such as delayed melatonin secretion and brain development, lead to alterations in circadian rhythms and sleep patterns. Consequently, adolescents exhibit a characteristic shift toward later bedtimes and wake-up times (1). Sufficient and high-quality sleep is crucial for adolescents’ physical and mental health, cognitive function, academic performance, and emotional regulation. Severe sleep issues can negatively impact both their physical and mental health (2). Sleep deprivation in adolescents is associated with declines in academic performance, increased emotional problems, and reduced cognitive function (3–10). Additionally, insufficient sleep is a risk factor for conditions such as obesity (11), hypertension (12), and cardiovascular diseases (13).

Nowadays, sleep deprivation is prevalent among adolescents. Surveys from the United States indicate that over 70% of high school students sleep less than the recommended minimum of 8 h. This proportion has continued to rise over the past decade, and the quality of sleep is also declining (14). According to the “2019 China Youth and Children’s Sleep White Paper,” which surveyed 42,339 students and their parents, the average sleep duration for adolescents aged 13 to 17 is less than 7 h. This is significantly shorter compared to previous years, and most teenagers are not getting enough sleep. In response to this issue, in March 2021, the Chinese government issued a notice to strengthen sleep management for primary and secondary school students (15). The notice emphasized the need for schools to balance work and rest times, reduce the academic workload, and include sleep status as part of physical health assessments. Later, in July 2021, the Chinese government issued another notice to further address sleep management. This was part of broader efforts to reduce the homework and extracurricular training burden on students, known as the “Double-Reduction” policy (16). The main reason behind the Chinese government’s “Double Reduction” policy is the heavy academic pressure faced by Chinese students. In response to this pressure, Chinese students often spend more time studying, either by choice or because they feel they have to. Schools sometimes extend class hours or add extra tutoring sessions. Students also attend after-school tutoring or arrive early for self-study. These practices often cut into their sleep time. To address this issue and give students more control over their schedules, the Chinese government launched the “Double Reduction” policy. These measures aim to ease academic pressure, give students more time for sleep, and support their overall physical and mental well-being.

Sleep duration, wake-up time, and bedtime are important measures for understanding sleep patterns (17). Most studies on teenage sleep rely on cross-sectional studies, that do not accurately assess continuous changes in sleep in the adolescent developmental period. Compared to the recall bias inherent in subjective questionnaires (18), objective measurement devices can accurately and objectively record relevant sleep parameters, providing new data sources and research methods for sleep studies.

The “Double Reduction” policy (July 2021) represents a major educational reform in China aimed at alleviating students’ academic burdens. Against this new policy backdrop, long-term, objective monitoring data on secondary students’ sleep patterns remains scarce. Therefore, this study aims to utilize wearable devices to initiate a three-year longitudinal investigation starting after policy implementation (December 2021), describing the evolving trajectories of secondary students’ sleep parameters.

## 2 Methods

### 2.1 Participants

Sample size calculation was performed *a priori* using G*Power software (version 3.1). The calculation was based on the primary outcome of the study, which was the night sleep duration. We used a two-tailed independent-samples t-test to compare the mean sleep duration between genders (male vs. female). The effect size was set to 0.15, representing a small effect based on pilot data and previous literature reporting small effect sizes in adolescent sleep research. The *α* error probability was set at 0.05, and the desired power (1-*β*) was 0.8 (19). The analysis indicated that a total sample size of 68 participants (34 per group) was required to detect such an effect. Considering a lost-to-follow-up rate of 20–30%, the final sample size selected for the study was 110 participants. In December 2021, we recruited 110 students from four grade7 at a secondary school in Nanjing, China, using a random cluster sampling method. Inclusion criteria: good physical health, no contraindications to exercise, and no psychiatric disorders. The experiment study ended in December 2023. During the three-year follow-up study, some participants withdrew from the experiment for reasons such as transferring schools, discomfort with wearing devices, and academic pressure. After analyzing the three-year data, the final group included 82 students: 40 boys and 42 girls. The dropout rate was 25.45%. The study was introduced to the school, teachers, and parents, and their consent was obtained. Parents signed an informed consent form. All participants were informed about the experimental plan, procedures, and related testing requirements. The study was approved by the Human Research Ethics Review Committee of the Nanjing Sports Institute (No. RT-2021-02), under the Declaration of Helsinki. The information of participants is presented in Table 1.

**Table 1:** Basic information of participants.

Characteristics	Male	Female
Age (years)	12.4 ± 0.5	12.3 ± 0.5
Height (cm)	164.8 ± 7.6	159.6 ± 6.0
Weight (kg)	58.2 ± 14.7	49.8 ± 10.3
BMI (kg/m^2^)	21.3 ± 4.6	19.4 ± 3.0

### 2.2 Device

This study collects sleep data from 82 students between December 2021 and December 2023 by Huawei Band 6. The three-year study was conducted for 2 weeks of sleep tracking and monitoring during the first semester of each academic year for middle school students in grade 7(G7), grade 8(G8) and grade 9(G9), from December 6, 2021, to December 21, 2021, November 15, 2022, to November 30, 2022, and November 22, 2023, to December 5, 2023, respectively. The studies all collected data during the students’ school year (not summer or winter vacation). In the study, subjects were required to wear the Huawei band for 24 h (except for the daily shower time, during which the bracelet was charged) and to upload the sleep data collected by the Huawei band to the HUAWEI Research cloud platform every day via a dedicated APP. The researchers then downloaded the data from the cloud platform and analyzed it.

The Huawei band integrates a diverse set of sensors and algorithms, enabling it to offer a thorough monitoring of both the user’s physiological and movement-related conditions. At its core, the wristband employs Photoplethysmography (PPG) to track autonomic functions, including heart rate and pulse wave patterns (20). In addition, through the incorporation of machine learning technologies (20, 21), the wristband can monitor sleep and wake cycles, leveraging motion detection sensors (e.g., accelerometers) alongside the observation of autonomic activities (20–22). Furthermore, advanced techniques such as cardiopulmonary coupling (CPC) (23) and heart rate variability (HRV) analysis (22) are applied to enhance the precision of sleep staging and quality assessments. These techniques, collectively, facilitate continuous and real-time health monitoring, coupled with comprehensive data analysis. Moreover, the accuracy of the wristband in sleep assessment has been verified through comparisons with in-lab video-polysomnography (23, 24).

### 2.3 Sleep data collection

The researchers downloaded the sleep data measured by the Huawei Band 6 from the cloud platform and analyzed it. The main indicators of sleep monitoring were light sleep duration, deep sleep duration, rapid eye movement (REM) duration, night sleep duration, daytime sleep duration, sleep onset time, and wake-up time. Night sleep duration is the time between sleep onset and awakening. Adequate sleep is defined as 8 hours of sleep at night. Study days and rest days are defined according to the school’s schedule, with study days Monday through Friday and rest days Saturday and Sunday in G7 and G8. Due to academic pressure, the school requires students to attend regular classes on Saturdays. This practice is customary in China, so this study designates Monday through Saturday as school days and Sunday as a rest day for ninth-grade students.

### 2.4 Statistical analysis

These data were analyzed using Excel and SAS JMP Pro 17.0.0. The Shapiro–Wilk test was used to assess the normality of the data. For normally distributed continuous data, the results are presented as mean ± standard deviation. Since we collected 3 years of data from students, recording approximately 14 consecutive days of sleep data each year, our method for calculating the mean and standard deviation of sleep characteristic metrics involved first determining the annual mean for each metric (e.g., sleep onset time, wake-up time, etc.) for each student. This yielded a single value per metric per student per year. We then calculated the annual mean and standard deviation for that metric based on the 82 values obtained from all 82 students. Categorical data were analyzed using frequency analysis and are expressed as percentages (%). A chi-square (*X*^2^) test was conducted to evaluate the distribution of students’ night sleep duration being sufficient (≥8 h) or insufficient (< 8 h). A mixed-effects model was employed to assess changes in sleep parameters among middle school students over the 3 years, across different genders and time types. In this model, fixed effects included the time (grade 7/8/9), gender (male/female), and time types (school days/rest days). The model also examined the interaction effects between time and gender, as well as between time and time types, to explore changes in sleep parameters among adolescents. Additionally, “student ID” was included as a random effect to account for individual differences. Post-hoc multiple comparisons were performed, and the Bonferroni correction was applied to control for the risk of false positives associated with multiple comparisons. The significance level for all tests was set at *p* < 0.05. Effect sizes were reported using partial ηsquared (*η*^2^*p*), with thresholds of 0.01, 0.06, and 0.14 representing small, medium, and large effect sizes, respectively.

## 3 Results

### 3.1 The sleep patterns of students over the 3 years

**Figure 1:**
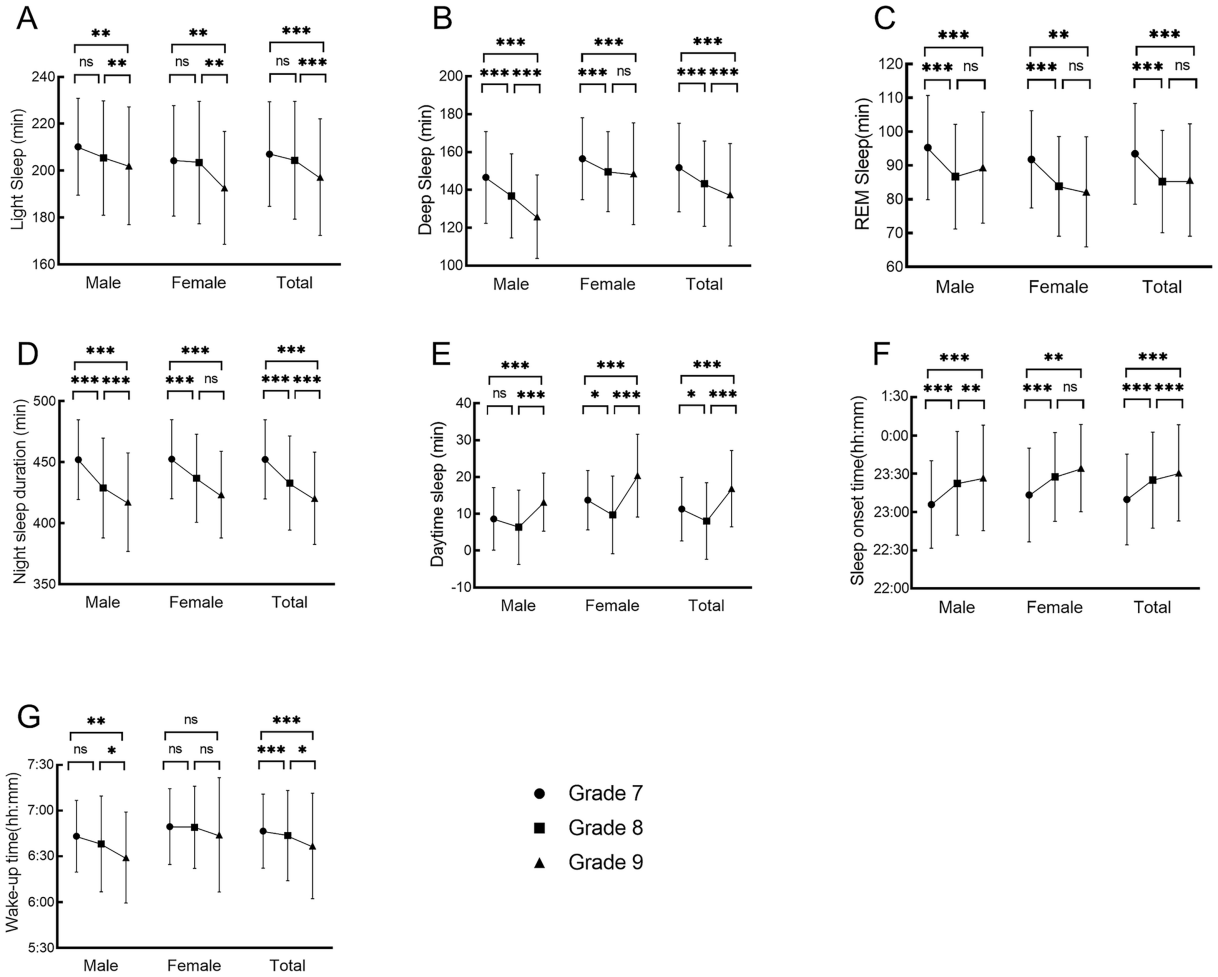
Changes in sleep characteristics of different genders in 3 years of middle school: **(A)** light sleep duration, **(B)** deep sleep duration, **(C)** REM duration, **(D)** night sleep duration, **(E)** daytime sleep duration, **(F)** sleep onset time, **(G)** wake-up time. *p < 0.05, **p < 0.01, ***p < 0.001.

There were significant changes in all sleep parameters of middle school students over the 3 years. Mixed-effects modeling showed that time had a significant effect on light sleep duration (*F* = 13.27, *p* < 0.001, *η*^2^*p* = 0.08), deep sleep duration (*F* = 47.75, *p* < 0.001, *η*^2^*p* = 0.11), REM duration (*F* = 35.79, *p* < 0.001, *η*^2^*p* = 0.14), night sleep duration (*F* = 50.30, *p* < 0.001, *η*^2^*p* = 0.11), daytime sleep duration (*F* = 49.09, *p* < 0.001, *η*^2^*p* = 0.18), wake-up time (*F* = 4.36, *p* = 0.0128, *η*^2^*p* = 0.04), and sleep onset time (*F* = 62.03, *p* < 0.001, *η*^2^*p* = 0.14). Gender had a significant effect on deep sleep duration (*F* = 6.29, *p* = 0.0133, *η*^2^*p* = 0.21) and daytime sleep duration (*F* = 9.28, *p* = 0.0027, *η*^2^*p* = 0.23), and there was no significant effect on other sleep parameters. The deep sleep duration for females in G8 and G9 was significantly longer than that of males (G8: *F* = 2.92, *p* = 0.0417, *η*^2^*p* = 0.25; G9: *F* = 5.04, P = <0.001, *η*^2^*p* = 0. 43). Additionally, females had significantly longer daytime sleep duration compared to males (G7: *F* = 3.05, *p* = 0.0283, *η*^2^*p* = 0.26; G9: *F* = 3.04, *p* = 0.0286, *η*^2^*p* = 0.26). The results are presented in Table 2 and Figure 1.

**Table 2:** Basic sleep characteristics for 3 years of middle school.

Variables	Grade	Light sleep (min)	Deep sleep (min)	REM sleep (min)	Night sleep (min)	Daytime sleep (min)	Sleep onset time (hh:mm)	Wake-up time (hh:mm)
Male	Grade7	210.2 ± 20.6	146.6 ± 24.2	95.3 ± 15.4	452.1 ± 32.6	8.6 ± 8.5	23:06 ± 0:36	6:42 ± 0:24
	Grade8	205.4 ± 24.4	136.8 ± 22.3	86.7 ± 15.5	428.9 ± 40.9	6.4 ± 10.1	23:18 ± 0:42	6:36 ± 0:30
	Grade9	202.1 ± 25.1	125.8 ± 22.1	89.4 ± 16.4	417.3 ± 40.4	13.2 ± 7.9	23:24 ± 0:42	6:30 ± 0:30
Female	Grade7	204.3 ± 23.5	156.5 ± 21.7	91.8 ± 14.4	452.5 ± 32.4	13.7 ± 8.1	23:12 ± 0:36	6:48 ± 0:24
	Grade8	203.5 ± 26.1	149.6 ± 21.1	83.8 ± 14.7	436.9 ± 36.1	9.7 ± 10.6	23:30 ± 0:36	6:48 ± 0:24
	Grade9	192.7 ± 24.1	148.6 ± 26.9	82.2 ± 16.3	423.4 ± 35.6	20.4 ± 11.2	23:36 ± 0:36	6:42 ± 0:36
Total	Grade7	207.1 ± 22.3	151.8 ± 23.3	93.5 ± 14.9	452.3 ± 32.4	11.3 ± 8.6	23:12 ± 0:36	6:48 ± 0:24
	Grade8	204.4 ± 25.1	143.3 ± 22.5	85.2 ± 15.1	432.9 ± 38.6	8.1 ± 10.4	23:24 ± 0:36	6:42 ± 0:30
	Grade9	197.3 ± 24.9	137.5 ± 27.0	85.7 ± 16.6	420.4 ± 37.9	16.9 ± 10.4	23:30 ± 0:36	6:36 ± 0:36

The interaction between time and gender was significant for deep sleep duration (*F* = 7.51, *p* = 0.0006, *η*^2^*p* = 0.06), but not for other sleep parameters. This indicates that the trends in deep sleep duration over the 3 years differed between males and females. Specifically, males experienced a year-by-year decrease in deep sleep duration, G8 and G9 are significantly reduced than G7 (G7 vs. G8: *F* = 4.67, *p* < 0.001, *η*^2^*p* = 0.12; G7 vs. G9: *F* = 9.18, *p* < 0.001, *η*^2^*p* = 0.25), and G9 is significantly reduced than G8 (G8 vs. G9: *F* = 4.91, *p* < 0.001, *η*^2^*p* = 0.13). Females showed a significant reduction in deep sleep duration in G8 and G9 compared to G7 (G7 vs. G8: *F* = 3.88, *p* = 0.0003, *η*^2^*p* = 0.10; G7 vs. G9: *F* = 4.16, *p* < 0.001, *η*^2^*p* = 0.11), but deep sleep duration remained similar between G8 and G9 (*F* = 0.79, *p* = 0.7076, *η*^2^*p* = 0.02). The results are presented in Table 2 and Figure 1.

### 3.2 Sleep patterns during school days and rest days over 3 years of middle school

**Figure 2:**
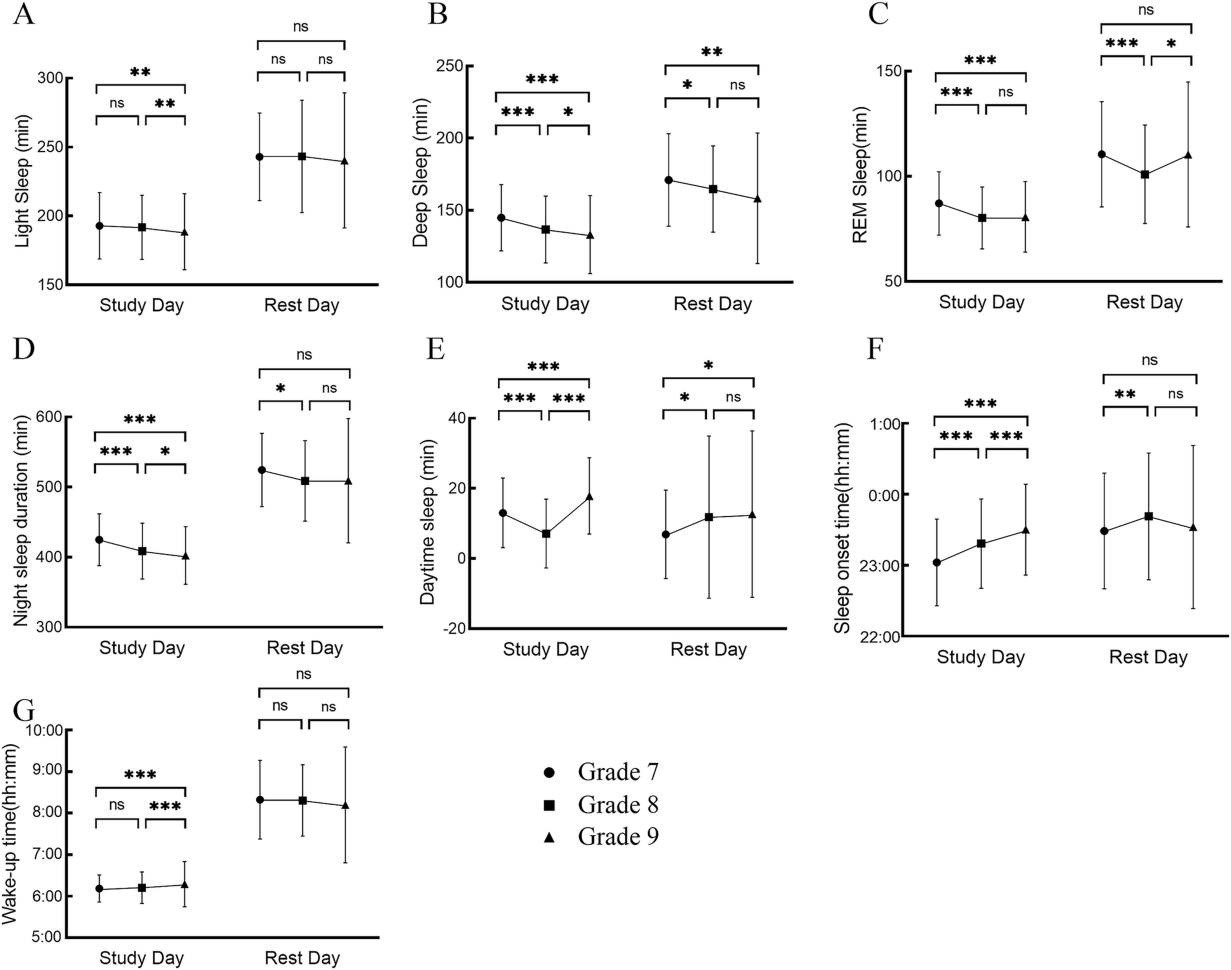
Changes in sleep characteristics of different time types in 3 years of middle school: **(A)** light sleep duration, **(B)** deep sleep duration, **(C)** REM duration, **(D)** night sleep duration, **(E)** daytime sleep duration, **(F)** sleep onset time, **(G)** wake-up time, *p < 0.05, **p < 0.01, ***p < 0.001.

Analysis using a mixed-effects model revealed that light sleep duration (*F* = 483.07, *p* < 0.001, *η*^2^*p* = 0.41), deep sleep duration (*F* = 211.67, *p* < 0.001, *η*^2^*p* = 0.27), REM sleep duration (*F* = 223.77, *p* < 0.001, *η*^2^*p* = 0.28), night sleep duration (*F* = 881.75, *p* < 0.001, *η*^2^*p* = 0.56) were longer on rest days than on school days. Wake-up time (*F* = 2560.92, *p* < 0.001, *η*^2^*p* = 0.95), and sleep onset time (*F* = 101.65, *p* < 0.001, *η*^2^*p* = 0.19) were later on rest days compared to school days. The interaction between time and time type was significant for daytime sleep duration (*F* = 21.60, *p* < 0.001, *η*^2^*p* = 0.12), wake-up time (*F* = 4.89, *p* = 0.0076, *η*^2^*p* = 0.06), and sleep onset time (*F* = 15.40, *p* < 0.001, *η*^2^*p* = 0.09). For daytime sleep duration on school days, there was a significant decrease from G7 to G8 (*F* = 7.33, *p* < 0.001, *η*^2^*p* = 0.16), a significant increase from G8 to G9 (*F* = 13.61, *p* < 0.001, *η*^2^*p* = 0.29), and a significant increase from G7 to G9 (*F* = 7.21, *p* < 0.001, *η*^2^*p* = 0.15). Further analysis revealed that daytime sleep duration was significantly longer on school days than on rest days for G7 and G8, while daytime sleep duration was significantly shorter on school days than on rest days for G9. On rest days, daytime sleep duration showed an increasing trend over the 3 years, G8 and G9 are significantly increased than G7 (G7 vs. G8: *F* = 2.69, *p* = 0.0198, *η*^2^*p* = 0.10; G7 vs. G9: *F* = 2.62, *p* = 0.0241, *η*^2^*p* = 0.12), and G9 is significantly increased than G8.(G8 vs. G9: *F* = 0.54, *p* = 0.8493, *η*^2^*p* = 0.02). Wake-up times on rest days were similar across the 3 years (*p* > 0.05), with a trend toward earlier wake-up times in G9 compared to G8 (*F* = 1.24, *p* = 0.4325, *η*^2^*p* = 0.05). On school days, wake-up times were similar between G7 and G8 but significantly later in G9 compared to G7 (*F* = 4.36, *p* < 0.001, *η*^2^*p* = 0.12). Sleep onset time on rest days were significantly later in G8 compared to G7 (*F* = 3.04, *p* = 0.0069, *η*^2^*p* = 0.12), with a non-significant trend toward earlier sleep onset time in G9 compared to G7 (*F* = 0.70, *p* = 0.7638, *η*^2^*p* = 0.01). On school days, sleep onset time gradually shifted later each year, G8 is significantly later than G7 (G7 vs. G8: *F* = 8.48, *p* < 0.001, *η*^2^*p* = 0.18) and G9 is significantly later than G8 (G8 vs. G9: *F* = 7.20, *p* < 0.001, *η*^2^*p* = 0.16). The results are presented in Table 3 and Figure 2.

**Table 3:** Sleep conditions of different grades on different time types.

Variables	Grade	Light sleep (min)	Deep sleep (min)	REM sleep (min)	Night sleep (min)	Daytime sleep (min)	Sleep onset time (hh:mm)	Wake-up time (hh:mm)
School day	Grade7	192.9 ± 24.1	144.8 ± 23.0	87.1 ± 15.0	424.9 ± 37.1	17.8 ± 10.9	23:00 ± 0:36	6:12 ± 0:18
	Grade8	191.9 ± 23.2	136.6 ± 23.2	80.7 ± 16.8	408.7 ± 39.9	13.0 ± 9.9	23:18 ± 0:36	6:12 ± 0:24
	Grade9	188.6 ± 27.6	133.1 ± 27.0	80.2 ± 14.7	402.4 ± 41.2	7.1 ± 9.8	23:30 ± 0:36	6:18 ± 0:30
Rest day	Grade7	243.2 ± 40.8	171.0 ± 32.1	110.5 ± 25.0	524.4 ± 52.3	6.9 ± 12.6	23:30 ± 0:48	8:18 ± 0:54
	Grade8	242.9 ± 31.8	164.7 ± 29.9	110.4 ± 34.5	509.1 ± 88.8	11.8 ± 23.1	23:42 ± 0:54	8:18 ± 0:54
	Grade9	240.3 ± 49.1	158.3 ± 45.2	101.0 ± 23.4	508.9 ± 57.4	12.7 ± 23.8	23:30 ± 1:06	8:12 ± 1:24

### 3.3 Sleep deprivation rates over the 3 years of middle school

**Figure 3:**
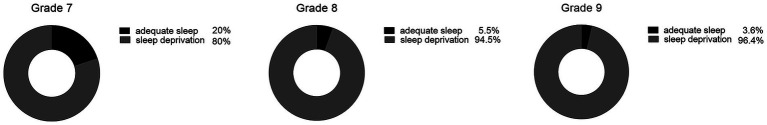
The proportion of sleep deprivation in 3 years of middle school.

In this study, night sleep duration of less than 8 hours was used as the criterion for determining sleep deprivation. *X*^2^ test showed that the incidence of sleep deprivation differed significantly across the first (80%), second (94.5%), and third (96.4%) years (*X*^2^ = 17.09, *p* = 0.0002, *η*^2^*p* = 0.0885). The proportion of students with adequate sleep decreased progressively over the 3 years, while the incidence of sleep deprivation among adolescents increased gradually (see Figure 3).

## 4 Discussion

Adolescent sleep is a critical determinant of overall health and well-being. This study thoroughly examines sleep patterns among Chinese adolescents. It aims to provide a scientific foundation for evaluating their sleep status and sleep sufficiency rates. Polysomnography (PSG) is considered the gold standard for measuring sleep. However, its operation is complex, costly, and time-consuming. These characteristics make it more suitable for small-scale comparative studies. Previous adolescent sleep studies have mostly used questionnaires, which are simple but may have recall bias. In contrast, this study used wearable technology, which can collect adolescent sleep parameters more objectively and accurately, providing reliable data support for a comprehensive understanding of adolescent sleep characteristics.

That study examined the sleep patterns of middle school students over 3 years (seventh, eighth, and ninth grades) after implementation of the policy. Findings indicate that students’ nighttime sleep duration decreased progressively with grade level, dropping from 452.3 ± 32.4 min in G7 to 420.4 ± 37.9 min in G9. Furthermore, students’ average sleep duration remained persistently below the recommended 8-h health standard (25). As students advance through the grades, the persistent decline in sleep duration is a cause for concern. The American Academy of Sleep Medicine recommends that adolescents aged 13 to 18 should regularly sleep 8 to 10 h to promote health (25). Therefore, this study classified adolescents who slept less than 8 h on average as sleep deprived. This study has shown that the prevalence of sleep deprivation among adolescents has increased over the years. This finding consistents with the results of a systematic review, which examined sleep duration among children and adolescents in 20 countries from 1905 to 2008 (26). It found that adolescents’ hours of sleep declined rapidly from year to year during this period, and the risk of developing sleep deprivation increased. Additionally, the night sleep duration of adolescents in this study was significantly lower than that of their peers in the United States (18), Malaysia (27), Australia (28), and Canada (29). The reported rate of sleep deprivation among students in each of the 3 years of middle school exceeded 80%, which was much higher than the national average (30). This means that Chinese middle school students still suffer from sleep deprivation.

The study found that over the 3 years, middle school students experienced a gradual reduction in light sleep, deep sleep, and REM sleep, while daytime sleep duration initially decreased and then increased. The significant reduction in daytime sleep during the second year may be attributed to the necessity of nucleic acid testing or other epidemic prevention measures implemented by schools during the COVID-19 pandemic, which occupied students’ nap time. This unavoidable confounding factor is associated with decreased daytime sleep duration. Research suggests that increasing nap time to make up for shorter night sleep can help students reach 8 h of total sleep, improving their cognitive function (31). Therefore, the reduction in daytime sleep for G8 students may have left them unable to compensate for sleep deprivation, resulting in issues such as daytime inattention. Additionally, the gradual decline in deep sleep duration from G7 to G8 may indicate a decrease in sleep quality. Deep sleep is considered a critical period for physical repair and recovery. This reduction reflects not only the overall decrease in total sleep duration but also a potential decline in the quality of sleep among adolescents.

In the study results, there was no significant difference in night sleep duration between males and females. However, gender had a significant effect on deep sleep duration and daytime sleep duration, with females having significantly more than males in both. Meanwhile, the interaction between time and gender on deep sleep duration was significant, and there were differences in the trends of deep sleep duration between males and females. This phenomenon among adolescents may be due to differences in physiological developmental rates and hormonal changes between genders. Male and female have different sleep patterns, including sleep quality and sleep structure. This is due to differences in thermoregulatory mechanisms and hormone levels between genders, which make biological rhythms inconsistent (32, 33). Females in this study had more hours of deep sleep and daytime sleep than boys, which is consistent with Roberts et al.’s study that women usually perform better in objective sleep indicators (34). Beyond physiological differences between genders, social factors such as gender roles, social opportunities, and lifestyle choices are hypothesized to influence sleep gender differences. However, due to the lack of explanatory variables in current research (e.g., adolescent mental health, pre-sleep procrastination, stress levels, dietary patterns, sleep hygiene, physical activity), coupled with the absence of questionnaire surveys to analyze sociocultural factors in this study, we cannot definitively elucidate the mechanisms underlying gender differences in adolescent sleep (35). This thus provides new directions for future research.

On school days, wake-up times were significantly earlier than on rest days, and sleep onset time gradually shifted later each year. This is because school schedules require students to wake up early on school days, while increasing academic pressure forces them to spend more time completing homework at night, leading to delayed sleep onset times. This schedule conflicts with adolescents’ natural sleep patterns. During adolescence, the secretion of melatonin, a hormone that regulates the sleep–wake cycle, is delayed at night compared to childhood. This delay results in later sleep onset times and a shift toward a “late to bed, late to rise” sleep pattern (36).

In this study, sleep onset time and wake-up time on rest days were later than on school days, and night sleep duration on rest days was significantly longer than on school days. This suggests that students tend to extend their sleep time on rest days to compensate for sleep deprivation during school days. This finding aligns with previous research, which conducted a cross-sectional study on the sleep habits of 735 adolescents, showing that adolescents experience insufficient sleep on school days due to academic pressure and heavy schedules, leading them to “compensate” by waking up later on rest days (37). However, this behavior may lead to more severe sleep rhythm disruption. This misalignment between biological and social clocks is defined as social jetlag (SJL), which serves as an indicator for assessing circadian rhythm disruption. Studies indicate that more severe SJL is associated with a higher risk of adverse health outcomes, including cognitive dysfunction, attention deficits, and mental disorders. The later sleep onset time on rest days may be attributed to parents allowing adolescents more leisure time, leading to increased use of electronic devices such as phones, computers, and tablets. The blue light emitted by these devices can suppress melatonin secretion, disrupt sleep patterns, and result in later sleep onset times (38). This delay in adolescents’ biological clocks conflicts with school schedule, which can lead to reduced hours of sleep at night potentially causing sleep problems and negatively affecting students’ physical and mental health (39). The study also found that daytime sleep duration was longer on school days than on rest days, which may be due to the fact that students compensate for insufficient night sleep on school days by taking naps to alleviate fatigue. When sleep deprivation occurs, the human homeostatic sleep drive increases, resulting in more fatigue and sleepiness during the day, which prompts adolescents to relieve fatigue by taking a nap (40). While napping may temporarily relieve fatigue caused by sleep deprivation, frequent napping can negatively affect nighttime sleep duration and quality, creating a vicious cycle (41).

Daytime sleep duration, wake-up time and sleep onset time showed significant interactions between time and time type (school day/ rest day). There was no statistically significant difference between the 3 years in wake-up time on rest days, but wake-up time on study days was delayed by about 6 min in the third year compared to the first year. However, the delay was only 6 min, failing to achieve a meaningful increase in sleep duration. At the same time, students continued to face substantial academic pressure and heavy homework loads, resulting in persistently delayed bedtimes on school days. Future schools should consider adjusting school schedules and incorporating education on the healthy use of smartphones. For instance, refrain from using smartphones, tablets, and other blue light-emitting devices at least 60 min before bedtime, blue light significantly suppresses melatonin secretion, disrupts circadian rhythms, and leads to difficulty falling asleep (42). It is recommended to delay school start times. Multiple studies have found that delaying the start of the school day by 30 min increases sleep duration by 11–20 min (43). This is not merely a simple addition of time; more crucially, it allows students’ sleep schedules to better align with their naturally delayed circadian rhythms, thereby improving sleep quality.

Limitations of this study. First and foremost is the inherent limitation of the research design. Due to the lack of baseline data prior to the implementation of the “Double Reduction” policy and the absence of a control group unaffected by the policy, we cannot causally attribute the observed sleep changes to the policy. Confounding factors such as the physiological circadian rhythm delay associated with adolescent development and the inherent increase in academic pressure with grade level may significantly influence sleep patterns. Therefore, the findings of this study should be interpreted as a valuable description of the sleep trajectories of middle school students during a specific historical period (post-“Double Reduction”) and within the policy context, rather than an evaluation of the policy’s effectiveness. Future studies employing quasi-experimental designs will be essential for more accurately assessing the policy’s impact.

## 5 Conclusion

This study reveals a trend of progressively shorter sleep duration among middle school students over 3 years, with persistent worsening of sleep deprivation issues. These findings indicate that, within the current policy context, middle school students’ sleep health continues to face significant challenges, influenced by complex factors. Recommendations include delaying morning class start times and strengthening sleep hygiene education for both parents and students to address this ongoing public health issue.

## Data Availability

The raw data supporting the conclusions of this article will be made available by the authors, without undue reservation.
